# Dynamic Characterization of Thermocouples under Double-Pulse Laser-Induced Thermal Excitation

**DOI:** 10.3390/s23052367

**Published:** 2023-02-21

**Authors:** Hongbo Yang, Chengxu Tu, Zhouxia Jia, Qingfu Meng, Jinghao Zhang, Jiaxiang Wang, Yalei Zhao, Chenbin Zhu, Fubing Bao

**Affiliations:** 1 Zhejiang Provincial Key Laboratory of Flow Measurement Technology, China Jiliang University, Hangzhou 310018, China; 2 Science and Technology on Reliability and Environment Engineering Laboratory, Beijing Institute of Structure and Environment Engineering, Beijing 100076, China; 3 Shanghai Institute of Measurement and Testing Technology, Shanghai 201203, China

**Keywords:** double pulse laser, thermocouple, dynamic calibration, time constant

## Abstract

This study investigated the dynamic characteristics of thermocouples by using double-pulse laser excitation for dynamic temperature calibration under extreme conditions. An experimental device was constructed for double-pulse laser calibration; the device uses a digital pulse delay trigger to precisely control the double-pulse laser to achieve sub-microsecond dual temperature excitation with adjustable time intervals. The time constants of thermocouples under single-pulse laser excitation and double-pulse laser excitation were evaluated. In addition, the variation trends of thermocouple time constants under different double-pulse laser time intervals were analyzed. The experimental results indicated that the time constant increases and then decreases with the decrease in the time interval of the double-pulse laser. A method for dynamic temperature calibration was established for the evaluation of the dynamic characteristics of temperature sensors.

## 1. Introduction

With the rapid development of aerospace and defense fields, dynamic temperature measurement has become crucial for applications such as the diagnosis of aero-engine combustion chambers [1], ground tests of hypercapnic engines [2], the heat flow test of hypersonic boundary layer transition [3], and the assessment of high-energy weapon destruction [4]. Thermocouples are extensively used for extreme temperature measurements owing to their advantages of high precision, large range, and small size. Since the last century, thermocouples have attracted considerable research attention. Existing thermocouples have response speeds on the microsecond scale. The data obtained through thermocouple measurements should be accurate and reliable for improving the quality of dynamic temperature measurements.

The dynamic response characteristics of thermocouples illustrate the relationship between the temperature of thermocouples and the temperature increments in the measured media. Existing thermocouples cannot immediately respond to changes in the measured transient temperature and require a certain period of time to reach thermal equilibrium; thus, the response speed of thermocouples is lower than the speed of temperature changes [5]. The time constant of a thermocouple is typically used to measure its response speed, which characterizes its ability to follow changes in the external temperature. The time constant of a thermocouple is related to structural dimensions, material properties, and the fabrication process. Because such parameters of a thermocouple are many and the structural shape of its temperature-sensing region cannot be accurately determined, the time constant of the thermocouple cannot be accurately obtained through theoretical calculations. Moreover, the time constant is also related to the operational and calibration environment. Therefore, the time constant of a thermocouple should ideally be determined through dynamic calibration tests.

Commonly used thermocouple calibration methods include the water bath/oil bath method, flame method, thermal wind tunnel method, excitation tube method, and pulsed laser method. Yang et al. [6] performed dynamic calibration experiments on Cu/CuNi thin film thermocouple by using the water bath method; the time constant of the sensor was 7.89 ms. Zhao et al. [7] developed a K-type thermocouple with a time constant of 847 ms, which was measured using a step signal generator through the fast sliding–type flame method. Yang et al. [8] analyzed the dynamic characteristics of thermocouples by using a modified thermal wind tunnel apparatus; the time constant of the thermocouples was 2 s. All the three aforementioned methods use physical ejection to introduce the thermocouple to high-temperature environments and to perform stepwise temperature excitation. Because a rapid temperature step signal cannot be easily generated using a physical ejection mechanical drive, the dynamic calibration requirements of existing high-speed thermocouples cannot be satisfied. The excitation tube method can be used to perform stepwise temperature measurements more effectively because of the fast propagation of the excitation wave. Li et al. [9] investigated the dynamic response of an E-type coaxial surface thermocouple to temperature excitation through the excitation tube method; the time constant of this sensor was 100 s. However, the experimental implementation is challenging because of the small step amplitude and short maintenance time of the excitation tube. The rate of changes in temperature of a laser is high, and the time to apply thermal excitation is in the order of nanoseconds; in addition, the parameters of a laser are highly tunable. Therefore, the laser method is most commonly used in high-speed thermocouple dynamic temperature calibration experiments. Armandas et al. [10] performed a pulsed-laser–based dynamic calibration test on a homemade Au-Ni thermocouple with a time constant of 10 μs. Li [11] used a pulsed laser to calibrate a probe-type fast thin-film thermocouple with a time constant of 6.6 μs. Penty Geraets et al. [12], Serio et al. [13], Qi [14], and Wang et al. [15] performed pulsed-laser–based dynamic performance tests on thermocouples and obtained time constant measurements in the order of microseconds, indicating that the laser method has great potential in the field of thermocouple calibration.

As discussed above, the current research on dynamic calibration mostly stays in the environment of single-pulse temperature variation, and little research has been carried out on the multi-pulse. However, the measurements using thermocouples were usually performed in environment of high dynamic temperature variation, such as measurement of high frequency dynamic temperature signals in aero-engine combustion chamber diagnosis [16] and dynamic process of high temperature inside gun firing [17]. To investigate the response characteristics of thermocouples under multiple temperature pulses, this study used a double-pulse laser as the thermal excitation source and established a thermocouple time constant measurement system based on the precise control of the double-pulse laser with a digital pulse delay trigger. The time constants of thermocouples under the single-pulse laser and double-pulse laser excitations were evaluated. Furthermore, the variation trend of the thermocouple time constants under different double-pulse laser time intervals was analyzed to verify the feasibility of the system here. The proposed method can be used to measure the time constants of thermocouples and to analyze their dynamic characteristics under extreme conditions.

## 2. Experimental Method

### 2.1. Thermocouple Dynamic Calibration Principle

**Figure 1:**
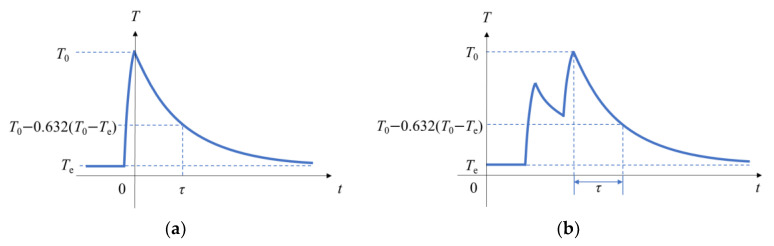
Schematic of definition of time constant of the thermocouple’s response to single-pulse laser (**a**) and double-pulse laser (**b**).

Thermocouples are typically considered first-order systems in dynamic calibration processes. The response of a thermocouple when it is excited by a unit pulse signal is illustrated in Figure 1a. The response signal can be expressed as follows:

(1) $$T-T_{e}=(T_{0}-T_{e})e^{-t/\tau }$$

where *T* denotes the output temperature of the thermocouple, *T*_0_ denotes the initial temperature of the thermocouple, *T*_e_ denotes the pulse equilibrium temperature of the thermocouple, *t* denotes the response time of the pulse excitation, and *τ* denotes the time constant of the thermocouple.

When *t* = *τ*,

(2) $$T=\frac{1}{e}(T_{0}-T_{e})+T_{e}=T_{0}-(1-\frac{1}{e})(T_{0}-T_{e})\approx T_{0}-0.632(T_{0}-T_{e})$$

When the thermocouple output response signal curve decayed to 63.2% of the entire temperature difference, the time required was equal to the time constant of the thermocouple.

The response of a thermocouple when it is excited by a double-pulse signal is illustrated in Figure 1b. Considering that the second laser pulse had a complete falling edge regardless of the length of the double-pulse time interval, the moment when the response curve reached the second peak was selected as the starting point, and the moment when the second falling edge of the response curve dropped to 63.2% of the temperature difference was selected as the end point to calculate the time constant of the thermocouple.

### 2.2. Experimental Device with a Double-Pulse Laser

**Figure 2:**
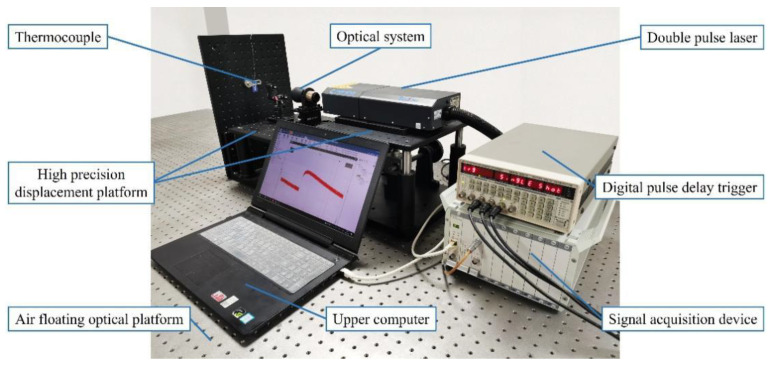
Dynamic calibration experimental device.

Figure 2 illustrates the proposed dynamic calibration system for thermocouples. The system comprises a double-pulse laser, a digital pulse trigger, an expanded beam focusing mirror, the thermocouple to be characterized, data acquisition equipment, and a host computer. The digital pulse delay trigger excites the double-pulse laser to produce two successive laser beams that thermally excite the temperature-sensing junction of the thermocouple. The response signal of the thermocouple is collected using the data acquisition equipment and then transmitted to the host computer for data processing and the calculation of the time constant.

**Figure 3:**
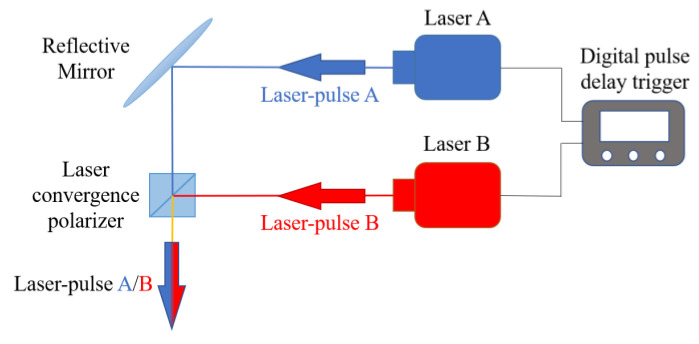
Standard double-pulse laser excitation signal generation system.

The design of the double-pulse laser standard excitation signal generation system is displayed in Figure 3. The system comprised a digital pulse delay trigger and a double-pulse laser. The double-pulse laser was constituted by two pulsed lasers A and B. The beam from laser A is reflected by a reflector to a laser beam polarizer and the beam from laser B is directed at the laser beam polarizer. The laser beam polarizer selectively enables the vibration of the beam in a certain direction by allowing the beam from laser A to pass through the laser beam polarizer and blocking and reflecting the beam from laser B. Thus, the beams from the two lasers combine to ensure that both pulsed laser beams irradiate the same temperature-sensing area of the microsecond temperature sensor.

The digital pulse delay trigger (Stanford-Research-Systems DG645) used in the experiments had a delay resolution of 5 ps, and the time control accuracy was in the order of picoseconds. The double-pulse laser (New Wave SOLO I) had a jitter of less than 1 ns and a time control accuracy in the nanosecond range. A nanosecond pulsed laser was used as the thermal excitation source, which was precisely controlled using a digital pulse delay trigger to successively generate two pulsed laser beams that were directed to the temperature-sensing area of the temperature sensor. Consequently, sub-microsecond dual temperature excitation was achieved with adjustable time intervals.

Because of the short duration of the pulsed laser excitation, the laser energy should be sufficiently high to satisfy the thermal excitation requirements of the thermocouple. The double-pulse laser used in this study had a maximum laser energy of 15 mJ and a repetition rate of 15 Hz. The laser energy can be changed by varying the laser parameters, and the required thermal excitation can be applied accordingly to the thermocouple. The laser was equipped with a beam-spreading focusing mirror, which facilitates the application of the laser radiation energy to the temperature-sensitive area of the thermocouple. The operation of the experimental device involved a high-precision displacement stage that enabled the precise positioning of the laser, the beam expander, and the thermocouple.

In this study, an SA1-K thermocouple was used for experimentation and analysis. The data acquisition equipment included the DH8302 dynamic signal test equipment and the Donghua test system, with a sampling rate of up to 1 M sample/s. The data were collected using the data acquisition equipment and transmitted to the host computer, which stored and processed the data and calculated the time constants.

## 3. Experimental Results

### 3.1. Calibration Experiment with Single-Pulse Laser Excitation

**Figure 4:**
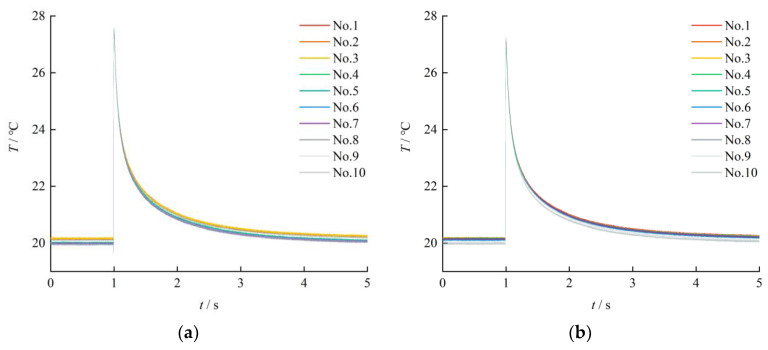
Thermocouple output response curve under single pulse laser excitation: (**a**) Laser A as the excitation source; (**b**) Laser B as the excitation source.

Single-pulse laser method has become one of the hottest methods of dynamic calibration of thermocouples, including S-type [18], N-type [19] and K-type [20] thermocouples. In order to facilitate comparison, the time constant of the SA1-K thermocouple under single-pulse laser excitation was measured by using the proposed double-pulse laser calibration system. The thermocouple was fixed on the test stand and positioned at the focal point of the lens by adjusting the high-precision displacement and the beam-expanding focusing optical path of the laser. The double-pulse laser was controlled using a trigger to emit only one laser beam, thereby realizing single-pulse laser excitation. The two pulsed lasers A and B constituting the double-pulse laser were used to excite the SA1-K thermocouple. The energy of the pulsed laser in the experiment was 13.64 mJ. The results of the calibration experiment are illustrated in Figure 4. The experiment was repeated 10 times with each laser; the lines in different colors in Figure 4 indicate different experimental groups. The moment when the response curve reached the peak was the starting point, and the moment when the response curve dropped to 63.2% of the temperature difference was the end point. The time constants of the thermocouple were calculated at these two moments, as summarized in Table 1.

The experimental results indicated that the average time constant of the SA1-K thermocouple under the excitation from laser A was 213.21 ms with a measurement standard deviation of 0.940 ms, and the average time constant of the SA1-K thermocouple under the excitation from laser B was 212.92 ms with a measurement standard deviation of 1.193 ms. The experimental results are in agreement with the time constant (*τ* < 0.3 s) provided by the SA1-K thermocouple manufacturer, and close to the time constant of the SA1-K thermocouple (*τ* = 0.217 s) measured by Ding et al. [21]. The thermocouple time constants measured using the two lasers of the double-pulse laser were identical, and the response curves of multiple calibrations were consistent. In addition, the experimental results had good repeatability.

### 3.2. Calibration under Double-Pulse Laser Excitation

**Figure 5:**
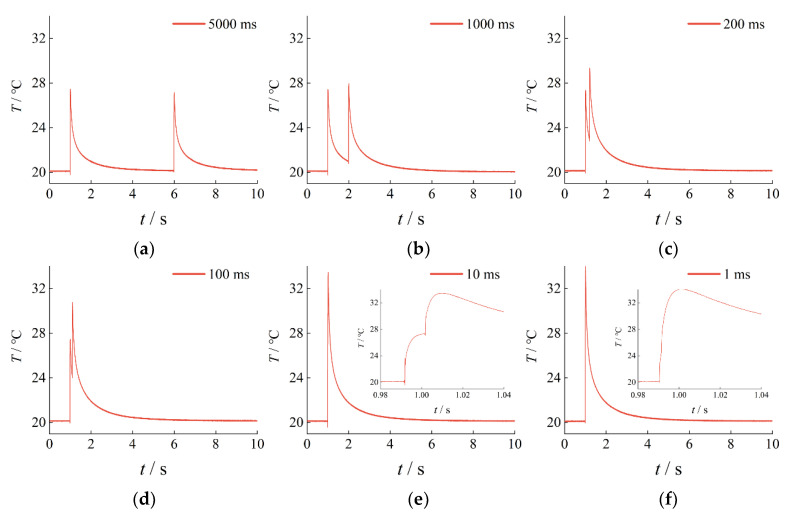
Thermocouple output response curves under double-pulse laser excitation over different time intervals: (**a**) Time interval = 5000 ms; (**b**) Time interval = 1000 ms; (**c**) Time interval = 200 ms; (**d**) Time interval = 100 ms; (**e**) Time interval = 10 ms; (**f**) Time interval = 1 ms.

**Figure 6:**
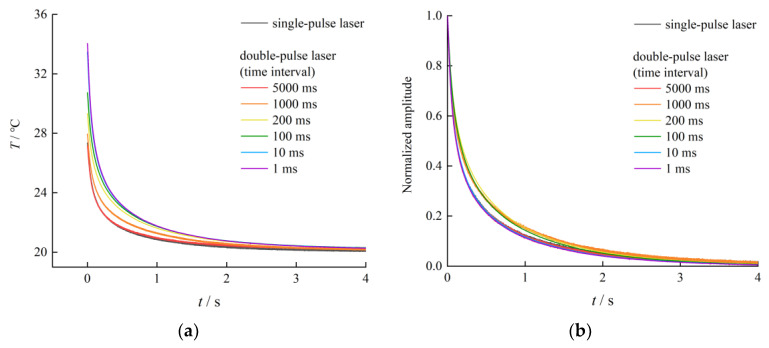
The comparison of output response curves under single and double pulse laser: (**a**) Comparison of falling edges of output response curves; (**b**) Comparison of the falling edge of the output response curve after amplitude normalization.

To analyze the dynamic response of a thermocouple under a double-pulse temperature excitation, a digital pulse delay trigger was used to control a double-pulse laser to successively emit two laser beams, each with an energy of 13.64 mJ. The beam from laser A irradiated on the thermocouple to generate a thermal excitation, and the output of the thermocouple was the first pulse response signal. The beam from laser B then irradiated on the thermocouple to generate another thermal excitation, and the output of the thermocouple was the second impulse response signal. Figure 5 displays the output response curves of the SA1-K thermocouple under double-pulse laser excitation over different time intervals. With the decrease in the time interval, the curve of the second impulse response signal of the output response curve moves closer to that of the first impulse response signal. When the time interval reached 1 ms, the second impulse response signal drowned out the first impulse response signal. The comparison of output response curves under single and double pulse laser is shown in Figure 6. The output response curve of the thermocouple is affected by the double-pulse laser excitation, and the effect of the double-pulse laser at different time intervals varies.

**Figure 7:**
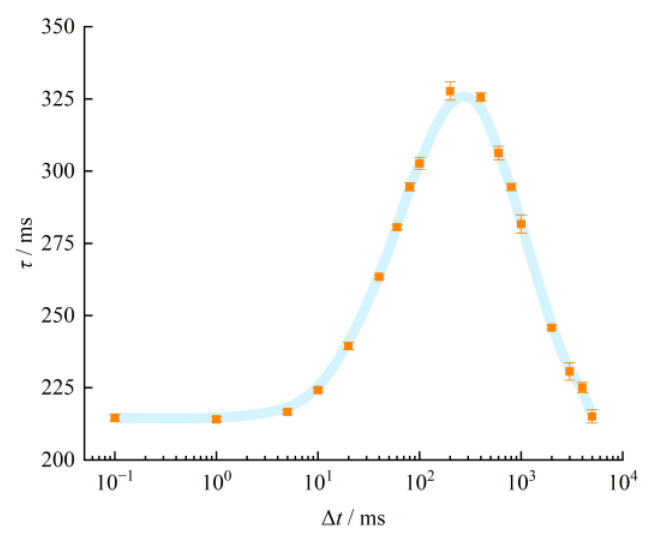
Experimental results under double-pulse laser excitation over different time intervals (Δ*t*).

The effects of different time intervals of the double-pulse laser excitation on the thermocouple time constant were quantitatively analyzed. The variations of the time constant of the thermocouple over the time interval of the double-pulse laser are illustrated in Figure 7. The orange dots with error bars indicate the experimentally measured time constants of the thermocouple under the excitation of the double-pulse laser at different time intervals. The blue line represents the trend line fitted through the orange data points. If the time interval was longer than 5000 ms, the time constant was largely unaffected and consistent with the experimental results of the single-pulse laser. When the time interval was between 200 and 5000 ms, the time constant increased with the decrease in the time interval. When the time interval was between 1 and 200 ms, the time constant decreased with the decrease in the time interval. When the time interval was reduced to 1 ms, the time constants obtained through the experiments performed using the single-pulse laser were consistent.

Under double-pulse laser excitation, the first pulse laser radiation was applied to the thermocouple temperature-sensitive junction. The temperature-sensitive junction absorbed the radiation heat, increasing the surface temperature of the thermocouple. When the laser excitation was removed, the thermocouple dissipated heat through thermal radiation and natural convection to the surrounding environment, and the surface temperature decreased rapidly. When the double pulse laser time interval was large and the second laser irradiated on the thermocouple, the temperature rise caused by the first laser decreased to approximately the ambient temperature. Meanwhile, the second pulse response curve of the thermocouple output was not affected by the first laser radiation. Therefore, the experimentally measured time constants were consistent with those obtained under the single-pulse laser excitation. When the time interval of the double-pulse laser decreased and the temperature rise caused by the first laser did not decrease to the equilibrium state, the second laser irradiated on the thermocouple immediately. The heat not dissipated from the thermocouple surface after the first irradiation affected the heat dissipation after the second laser irradiation, causing the second pulse response curve to decrease gradually. By contrast, the double-pulse laser excitation gradually tended to the single-pulse laser excitation when the first laser and the second laser gradually approached each other. The experimental results of the double-pulse laser excitation gradually tended to the results of the single-pulse laser excitation. Accordingly, when the time interval of the double-pulse laser decreased, the time constant of the thermocouple first increased gradually and then decreased gradually to be consistent with the experimental results obtained under the single-pulse laser excitation.

## 4. Conclusions

This study established a thermocouple time constant measurement system with double-pulse laser excitation. To analyze the dynamic characteristics of thermocouples under multi-pulse temperature, the time constants of SA1-K thermocouples under single-pulse laser excitation and double-pulse laser excitation were measured, respectively. The experiments revealed that the time constants of the thermocouple under single-pulse laser excitation were 213.21 and 212.92 ms for two double-pulse lasers A and B, respectively. The experimental results were consistent. The variation trends of thermocouple time constants under different double-pulse laser time intervals were analyzed. The thermocouple time constant measured under double-pulse laser excitation tends to increase and then decrease with the decrease in the double-pulse laser time interval. The inflection point is reached when the time interval is about 200 ms, which is close to the thermocouple time constant under single-pulse laser excitation. When the time interval was greater than 5000 ms or less than 1 ms, the time constant measured under double-pulse laser excitation was equal to that measured under single-pulse laser excitation. We thus demonstrated a dependence of the thermocouple’s dynamic characteristics on the number of laser pulses. This study may serve to create a new thought process for evaluating the dynamic characteristics of thermocouple under high dynamic temperature variation environment.

## Figures and Tables

**Table 1:** Thermocouple time constants under single pulse laser excitation.

No.	1	2	3	4	5	6	7	8	9	10
$\tau_{A}$/ms	212.9	214.3	213.9	213.3	213.2	212.3	212.4	212.7	212.1	215.0
$\tau_{B}$/ms	213.4	212.5	212.6	215.6	212.2	211.4	212.2	214.1	212.9	212.3

## Data Availability

The data that support the findings of this study are available from the corresponding author upon reasonable request.
